# Robust multivariate regression controlling false discoveries for microbiome data

**DOI:** 10.1093/bioinformatics/btaf506

**Published:** 2025-09-17

**Authors:** Gianna Serafina Monti, Meritxell Pujolassos, Malu Calle Rosingana, Peter Filzmoser

**Affiliations:** Department of Economics, Management and Statistics, University of Milano-Bicocca, Milan 20126, Italy; Bioscience Department, Faculty of Sciences, Technology and Engineering, University of Vic, Central University of Catalunya, Vic 08500, Spain; Bioscience Department, Faculty of Sciences, Technology and Engineering, University of Vic, Central University of Catalunya, Vic 08500, Spain; Institut de Recerca i Innovació en Ciències de la Vida i de la Salut, Catalunya Central (IRIS-CC), Vic, Barcelona 08500, Spain; Institute of Statistics & Mathematical Methods in Economics, Vienna University of Technology, Vienna 1040, Austria

## Abstract

**Motivation:**

Understanding how bacterial species relate to clinical health indicators can reveal microbiome signatures of disease, offering insights into conditions such as obesity or liver disease. However, analyzing such data requires methods that address compositionality, high dimensionality, sparsity, and outliers.

**Results:**

We tackle the challenge of identifying microbiome components linked to health indicators through a robust multivariate compositional regression model. Our method addresses the high dimensionality, sparsity, and compositional nature of microbiome data while maintaining control of the false discovery rate (FDR). By incorporating outlier robustness and a derandomization step, we enhance the stability and reproducibility of results, surpassing current techniques like the Multi-Response Knockoff Filter (MRKF). In simulation studies, our method outperforms MRKF in terms of FDR control, power, and robustness. In real data applications, it leads to valuable biological insights, such as identifying microbial species associated with specific clinical parameters.

**Availability and implementation:**

Software in R code format, along with synthetic data example illustrations and comprehensive documentation, is available at https://github.com/giannamonti/RobMReg.

## 1 Motivation

The intestinal microbiome is vital for host health and metabolism, with growing evidence that shifts in microbial communities reflect changes in health. This has increased interest in identifying microbiome signatures associated with clinical outcomes, including obesity, liver disease, and cancer. A primary goal is to find bacterial species associated with specific health indicators. Analyzing microbiome data involves several statistical challenges. The data are compositional—meaning important information resides in the ratios between taxa—high-dimensional, and sparse, since only a small subset of features usually relate to outcomes. Additionally, multidimensional outliers can skew results, and multivariate outcomes are common, particularly when multiple clinical parameters or phenotypes are measured simultaneously. While existing methods address some of these aspects—such as false discovery rate (FDR) control or compositionality—they usually focus on univariate settings and are not robust to outliers. Therefore, a unified framework is needed that can jointly handle compositional predictors, multiple correlated outcomes, and control for contamination by outliers, while also controlling the FDR. To address this gap, we propose a robust multivariate regression method designed for compositional covariates, incorporating both FDR control and a derandomization step to enhance result stability and reproducibility. Specifically, our method extends the Multi-Response Knockoff Filter (MRKF) of Srinivasan *et al.* (2023), improving it with robust estimation techniques and principled derandomization via the e-BH procedure (Wang and Ramdas 2022, Ren and Barber 2024). In this context, the regression problem is modeled as a multivariate model, where $Y={\left(Y_{1},\ldots ,Y_{q}\right)}^{T}$ represents multiple clinical or phenotypic outcomes, and the predictors are high-dimensional microbiome components. Secondary response variables—such as phenotypes in a genetic context—can also be analyzed to understand better their association with microbiome composition and their relationship with the primary outcome. Using multivariate models enables simultaneous inference and overcomes the limitations of univariate approaches, which test associations separately and require multiple testing corrections (see e.g. Wen and Lu 2022). Our proposed method provides improved power, robustness, and reproducibility for high-dimensional microbiome studies under FDR control.

The outline of the paper is as follows. Section 2 reviews the classical multivariate regression method involving compositional covariates and presents the proposed robust version. Further, it details how the MRKF is robustified and extended to a version for FDR control using the concept of *e*-values. The simulation results are described in Section 3 to evaluate the numerical performance of the proposed method in comparison with the non-robust method. Section 4 presents an application to real microbiome data, and the final Section 5 concludes.

## 2 Materials and methods

The computational methods we will present in this section are an adaptation of the robust multivariate regression method with covariance matrix estimation presented by Chang and Welsh (2023), adjusted for the case of compositional covariates that we call *alr*RMCL. The optimization algorithm we present is similar to, but not the same as, the algorithm of Chang and Welsh (2023). Important modifications have been introduced to make it compatible with the compositional nature of the data and to improve its efficiency. Moreover, a derandomized robust knockoff filter (Barber and Candès 2015, Ren and Barber 2024) that controls the FDR by adding “knockoff” variables to the regression is proposed.

It has been shown that the knockoff-based methods also have e-value interpretations (Ren and Barber 2024). Based on this observation, we applied an e-BH procedure for multiple testing problems that control the FDR under arbitrary dependence (Wang and Ramdas 2022).

### 2.1 Preprocessing

Let $Y={\left(Y_{1},\ldots ,Y_{q}\right)}^{T}$ be the vector of *q* responses related to the same disease, and they are supposed to be correlated.

Let $W={\left(W_{1},\ldots ,W_{p+1}\right)}^{T}$, with $W_{\kappa }>0$ for all κ, be a vector of absolute abundances of the (p+1) different taxa, i.e. operational taxonomic units. The microbiome datasets generated by high-throughput sequencing of 16S rRNA gene amplimers, metagenomes, or metatranscriptomes are typically considered compositional because the total number of counts within a sample is irrelevant. Indeed, they have an arbitrary total imposed by the instrument (Gloor *et al.* 2017). The data of interest are instead the relative values of the read counts, which makes the dataset inherently compositional. This is precisely the only useful information we want to extract from the microbiome’s composition. Experimental limitations, such as variations in the library size corresponding to the total count in each sample, do not allow for a direct analysis of the count data; therefore, the relative abundances of each taxon must be considered as a datum.

Let $X={\left(X_{1},\ldots ,X_{p+1}\right)}^{T}$ with $X_{\kappa }=W_{\kappa }/\sum_{\ell =1}^{p+1}W_{\ell }$ for κ=1,…,p+1, the normalized vector, to eliminate the effect of the sample totals. We observe that the (p+1) components of *X* are positive, thus $X_{\kappa }>0$, and are subject to the unit sum constraint $\sum_{\kappa =1}^{p+1}X_{\kappa }=1$, namely $X\in S^{p+1}$, i.e. the unit simplex. We assume we have measured the microbiome abundances related to the (p+1) taxa from *n* samples. Let X be the compositional matrix of dimension n×(p+1), where each row contains all the relative information among the components.

The log-ratio approach is popular for extracting and analyzing relative rather than absolute information (Aitchison 1982). A first step is to represent the data by using a specific transformation, where we propose to use the additive logratio (*alr*) transformation, defined as

$$Z={\left(Z_{1},\ldots ,Z_{p}\right)}^{T}:={\left(\text{log}\;\frac{X_{1}}{X_{p+1}},\ldots ,\;\text{log}\;\frac{X_{p}}{X_{p+1}}\right)}^{T},$$

where $Z\in R^{p}$ is the microbiome feature vector, and $X_{p+1}$ is the *reference frame* (Morton *et al.* 2019, Brill *et al.* 2022). The choice of the reference frame is a crucial point, as all the subsequent analyses will depend on it (Greenacre *et al.* 2021). On the other hand, the alr transformation allows for a clear model interpretation in terms of variable selection in a sparse setting. However, caution should be exercised when interpreting the regression coefficients, as additive log-ratios should be understood as increasing one component relative to all others, rather than only with respect to the chosen reference, as pointed out in Coenders and Pawlowsky-Glahn (2020).

It seems convenient that the reference component is not differentially abundant across the samples. Furthermore, although compositional data must be interpreted in terms of relative information, from an operational perspective, the chosen reference component should not be strongly associated with the response variables. To guide this choice, we suggest performing a robust test of association (e.g. Spearman’s or Kendall’s rank correlation) between each potential reference component $X_{\kappa }$ and the response vector *Y*.

Note that here the normalization of the composition to unit sum is essential, while it is irrelevant for the alr transformation. The component with the lowest maximum association measure (Alfons *et al.* 2017) will be the suitable candidate for the choice of reference. Furthermore, as observed, e.g. in Brill *et al.* (2022), domain knowledge should also be considered when choosing the reference, particularly in cases with multiple options. In addition, alternative approaches have been proposed in the literature to identify suitable reference taxa in a data-driven manner, such as RioNorm2 (Ma *et al.* 2020), which uses a network-based normalization strategy to detect relatively invariant taxa across samples and conditions. While not specifically designed for robustness, such methods can be useful complements in preprocessing pipelines, especially when no clear biological guidance is available. Other methods, such as RAIDA (Sohn *et al.* 2015), ANCOM (Mandal *et al.* 2015), and Omnibus (Chen *et al.* 2018), are examples of differential abundance testing procedures designed explicitly for microbiome data. These methods address common challenges in microbiome sequencing, such as compositional heterogeneity, zero inflation, overdispersion, and outliers, although they often rely on distributional assumptions or non-robust frameworks. Our robust knockoff-based approach, combined with alr transformation under sparsity assumptions, offers a complementary and interpretable alternative in high-dimensional settings.

Hereafter, to fix the notation, we use index *j* as the index of the microbiome features (j=1,…,p), *i* as the index of the sample (i=1,…,n), and *h* as the index of the response variables (h=1,…,q).

### 2.2 Multivariate regression with covariance estimation

To link the microbial features to the response variables, we consider a multivariate regression model, which could be expressed in matrix form as

(1) $$\underset{n\times q}{Y}=\underset{n\times p}{Z}\underset{\;p\times q}{B}+\underset{n\times q}{E},$$

where Y is the response matrix related to *q* continuous outcome variables, Z is the design matrix of microbial (alr transformed) features, B is the regression coefficients matrix, and E is the error matrix, with rows $\varepsilon_{i}\overset{\mathrm{iid}}{∼}N_{q}(0,\underset{q\times q}{\Sigma })$ for some unknown positive-definite covariance matrix Σ.

W.l.o.g., we can assume that Y and Z are centered, which allows us to remove the intercept from the model (1).

To address the estimation difficulties of the model (1), a relatively simple solution is to ignore the correlation structure of the errors and obtain a maximum likelihood estimate of B minimizing the Gaussian negative log-likelihood up to a constant,

(2) $$\text{tr}[{\left(Y-\mathrm{ZB}\right)}^{T}(Y-\mathrm{ZB})\Omega ]-n\;\text{log}\;|\Omega |,$$

where $\Omega =\Sigma^{-1}$ is the precision matrix, tr[·] denotes the trace of a matrix, and |Ω| is its determinant. It can be shown that the maximum likelihood solution of (2) for B coincides with the least squares solution, i.e. ${\hat{B}}_{\text{OLS}}={\left(Z^{T}Z\right)}^{-1}Z^{T}Y$. This multivariate problem can be viewed as a series of *q* univariate least squares problems, one for each response variable, and each providing the estimate of the *h*th column of the matrix B.

However, as pointed out in Rothman *et al.* (2010) and Lee and Liu (2012), fitting separate models for each response ignores the correlation structure among outcomes, potentially leading to inefficient estimation, unstable variable selection, and reduced statistical power. In contrast, multivariate approaches can exploit shared information across responses, improving prediction performance. Thus, to tackle the multiple-response regression problem with the two simultaneous goals of parameter estimation and variable selection, Rothman *et al.* (2010) proposed a penalized normal likelihood framework, with a lasso penalty (Tibshirani 1996) to promote coefficient sparsity and account for the high-dimensional setting. We will refer to their approach with the acronym MRCE:

(3) $$\begin{array}{c} ({\hat{B}}_{\text{MRCE}},{\hat{\Omega }}_{\text{MRCE}})=\\ \underset{B,\Omega }{\text{argmin}}\{\text{tr}[{\left(Y-\mathrm{ZB}\right)}^{T}(Y-\mathrm{ZB})\Omega ]-n\;\text{log}\;|\Omega |\\ +\lambda_{1}\sum_{j^{\prime }\neq j}|\omega_{j^{\prime }j}|+\lambda_{2}\sum_{j=1}^{p}\sum_{h=1}^{q}|\beta_{jh}|\}, \end{array}$$

where $\omega_{j^{\prime }j}$ and $\beta_{jh}$ are the entries $\left(j^{\prime },j\right)$ and (j,h) of Ω and B, respectively, and $\lambda_{2}\geq 0$ and $\lambda_{1}\geq 0$ are tuning parameters to control sparsity in B and Ω. The simultaneous estimation of the regression coefficients and the covariance structure of the MRCE is performed via a fast approximate algorithm that utilizes an alternating estimation scheme, where one matrix is held fixed at each step. When Ω is fixed, the solution for B can be efficiently obtained using the cyclical coordinate descent algorithm (Friedman *et al.* 2007). Conversely, when B is fixed, the solution for Ω can be determined using the graphical lasso algorithm (Friedman *et al.* 2008). For more details on MRCE, see Rothman *et al.* (2010). Note that the $\ell_{1}$ penalty on both B and Ω is compatible with the assumption of sparsity in the regression coefficients, meaning that only a small portion of the covariates can predict the responses, providing interpretation. It is also consistent with the assumption that only some response variables are correlated with each other, improving prediction performance.

It is well known that estimation methods based on likelihood maximization are highly sensitive to the presence of outliers in the data. To address this issue, Chang and Welsh (2023) proposed a robust alternative to MRCE, namely the robust multivariate lasso regression with covariance estimation (hereafter referred to as RMLC).

In this contribution, we adapt RMLC to the context of compositional data analysis, introducing key modifications to the original algorithm. We refer to our method as *alr*RMLC to highlight its close connection to the RMLC algorithm and our choice of the *alr* transformation to accommodate the compositional nature of the microbiome.

The objective function of the *alr*RMLC is defined as

(4) $$\begin{array}{c} ({\hat{B}}_{\mathrm{alr}\text{RMLC}},{\hat{\Omega }}_{\mathrm{alr}\text{RMLC}})=\\ \underset{B,\Omega }{\text{argmin}}\{2\sum_{i=1}^{n}\sum_{h=1}^{q}\rho ({\left[(Y-\mathrm{ZB})\Omega^{1/2}\right]}_{ih})-\\ n\;\text{log}\;|\Omega |+\lambda_{1}\sum_{j^{\prime }\neq j}w_{1,j^{\prime }j}|\omega_{j^{\prime }j}|+\lambda_{2}\sum_{j=1}^{p}\sum_{h=1}^{q}w_{2,jh}|\beta_{jh}|\}, \end{array}$$

where ${\left[\;\;\right]}_{ih}$ defines the element (i,h) of a matrix, $\omega_{j^{\prime }j}$ and $\beta_{jh}$ are the entries $\left(j^{\prime },j\right)$ and (j,h) of Ω and B, respectively, and $\lambda_{2}\geq 0$ and $\lambda_{1}\geq 0$ are tuning parameters to control sparsity in B and Ω as in (3). In addition to the objective function (3), two adaptive weight systems have been introduced in function (4), namely $w_{1,j^{\prime }j}$ and $w_{2,jh}$, to allow different penalties for each entry of Ω and B. A standard choice (Zou and Zhang 2009, Lee and Liu 2012) is $w_{2,jh}=\frac{1}{\left|{\tilde{\beta }}_{jh}\right|}$, where ${\tilde{\beta }}_{jh}$ is the OLS estimator in the low-dimensional setting (i.e. when p<n) or the $\ell_{2}$ (ridge) estimator in the high-dimensional case (p>n). Chang and Welsh (2023) suggested to replace ${\tilde{\beta }}_{jh}$ with its robust counterpart resulting from *q* separate MM-ridge regressions (Maronna 2011) to account for contamination in the data. Note that in the objective function (4), different levels of shrinkage are applied to the regression coefficients and the precision matrix by introducing adaptive weights $w_{1}$ and $w_{2}$ in the same fashion as an adaptive lasso regularization method (Zou 2006). Although we provided a fully general formulation of the problem in (4), in the implementations we set all weights $w_{1,j^{\prime }j}$ and $w_{2,jh}$ to 1 to avoid making the problem more computationally demanding.

In the objective function (4), ρ is a scalar symmetric robust loss function. In the following, we will use Tukey’s biweight loss, defined as

(5) $$\rho_{d}(x)=\{\begin{array}{cc} \frac{d^{2}}{6}\{1-{\left[1-{\left(\frac{x}{d}\right)}^{2}\right]}^{3}\} & \text{if}\;|x|\leq d\\ \frac{d^{2}}{6} & \text{if}\;|x|>d \end{array},$$

where *d* is a positive tuning constant to control the level of robustness. A common choice is d=4.685, which yields approximately 95% asymptotic efficiency under normality. Tukey’s biweight loss is a preferable choice as it provides robustness against outliers in the responses as well as high-leverage points or outliers in the covariates. Note that MRCE is a special case of *alr*RMLC, when ρ corresponds to the squared loss and $w_{1,j^{\prime }j}\equiv 1$, $w_{2,jh}\equiv 1$.

To numerically optimize the objective function (4) for the joint estimation of B and Ω, we propose the *alr*RMLC algorithm, inspired by Chang and Welsh (2023). This procedure consists of three steps and incorporates a 2-fold accelerated proximal gradient (APG) method. First, we optimize B for a specified precision matrix Ω via robust multivariate Lasso (RML); second, we estimate Ω for a given B through a robust extension of the graphical Lasso (Rglasso); third, the first two steps are iterated until convergence. To reduce computational cost, we adopt a fast approximation in which the outer iteration is performed only once, as summarized in Algorithm 1.

Algorithm 1 Algorithm for solving *alr*RMLC (a fast approximation)

** Data**: n×q response matrix Y; n×p microbial features design matrix Z, $B_{\mathrm{ini}}$, $\lambda_{1}$, $\lambda_{2}$.

** Output**: $\hat{B}$ and $\hat{\Omega }$

** Procedure**

** **1. given $\Omega_{0}$ compute ${\hat{B}}_{0}=\hat{B}(\Omega_{0})$ using RML

** **2. given $B_{0}={\hat{B}}_{0}$ compute $\hat{\Omega }=\hat{\Omega }(B_{0})$ using Rglasso

** **3. recompute $\hat{B}=\hat{B}(\hat{\Omega })$ using RML.

Details on computational algorithms are reported in Section 1, available as supplementary data at *Bioinformatics* online.

### 2.3 Multi-response Knockoff filter

In the context of the microbiome, one must contend with the curse of dimensionality, where the number of covariates far exceeds the sample size. This often results in the selection of numerous false positives—i.e. irrelevant variables—compromising the reproducibility of results.

To address this issue, the compositional knockoff filter (Srinivasan *et al.* 2021) has been proposed, leveraging a fixed-X design (Barber and Candès 2015). Later, in Srinivasan *et al.* (2023), a model-X knockoff filter (Barber and Candès 2019) was introduced to control the false discovery proportion in high-dimensional settings without requiring assumptions on the conditional distribution of the responses. In Monti and Filzmoser (2024), a two-step robust compositional knockoff filter for compositional covariates based on the recycled fixed-X knockoff procedure (Barber and Candès 2015, 2019) was considered to robustify the algorithm proposed by Srinivasan *et al.* (2021). While Srinivasan *et al.* (2021) and Monti and Filzmoser (2024) focus on univariate regression settings, Srinivasan *et al.* (2023) addresses the multivariate response case. In this contribution, we propose the model-X knockoff filter for robust multivariate regression with covariance estimation as an effective method to control the FDR of the selected covariates, which serves as a robust counterpart of the Multi-Response Knockoff Filter (MRKF) of Srinivasan *et al.* (2023).

The MX problem explored by Candès *et al.* (2018) can be imagined as testing, for each j∈[p·q]={1,…,pq}, whether $Z_{j}$ is related to at least one $Y_{h}$ given all other variables except $Z_{j}$ (denoted as $Z_{-j}=\{Z_{1},\ldots ,Z_{p}\}\setminus Z_{j}$). In other words, the goal is to determine whether each of the following [p·q] null hypotheses

(6) $$H_{0,\;j}:Y⊥⊥Z_{j}|Z_{-j}$$

holds. The MX knockoff filter is conceived to test $H_{0,\;j}$ in (6) for all j∈[p·q], and indeed it should be noted that each feature $Z_{j},j=1,\ldots ,p$, may be potentially related to each response variable $Y_{h}$, h=1,…,q. Ideally, the goal of the selection procedure is to identify the smallest subset of the features Z for which $H_{0,\;j}$ is not true.

A variable $Z_{j}$ is considered non-null, i.e. important, if $H_{0,\;j}$ is not true, indicating a feature with a nonzero effect on at least one response variable. As the number of hypotheses and discoveries may be large, we want to test $H_{0,\;j}$ in (6) while controlling the false discovery rate (FDR), i.e. the expected proportion of false positives—null hypotheses that are true but are incorrectly rejected—among the total number of selected features, i.e. all rejected hypotheses,

(7) $$\text{FDR}:=E[\frac{\left|\hat{S}\cap H_{0}\right|}{\max\{|\hat{S}|,1\}}],$$

where, with a slight abuse of notation, $\hat{S}$ and $H_{0}$ correspond to the set of indices related to the rejected nulls and the true null, respectively.

A selection rule controls the FDR at level α∈(0,1) if its FDR is guaranteed to be at most α, regardless of the values of the coefficients B.

Details on the multi-response knockoff filter are reported in Section 2, available as supplementary data at *Bioinformatics* online.

We perform the robust multivariate regression with covariate estimate using the *alr*RMLC algorithm on the augmented dataset $\left(Z,\tilde{Z},Y\right)$, which includes 2*p* predictors and *q* responses.

Thus, following the knockoff framework, to identify the relevant variables obtained from the variable selection procedure described in Algorithm 1, we compute the feature importance statistics as the lasso coefficient difference after tuning the regularization via cross-validation,

(8) $$W_{j}=|{\hat{\beta }}_{j}|-|{\hat{\tilde{\beta }}}_{j+p}|,\;j\in [p\cdot q],$$

which compares the estimated coefficient of the original feature $Z_{j}$ for the *h*th response ${\hat{\beta }}_{j}$ with those of knockoff features ${\hat{\tilde{\beta }}}_{j+p}$. The importance statistic (8) has the property that swapping $Z_{j}$ with ${\tilde{Z}}_{j}$ flips the sign of $W_{j}$, so that larger positive values of $W_{j}$ indicate that $Z_{j}$ is a “true” signal, i.e. $Z_{j}$ has a nonzero effect on one response. The final set of selected features is given by

(9) $$\begin{array}{c} {\hat{S}}_{\text{kn}}:=\{j,W_{j}\geq T_{\alpha }\},\text{where}\\ T_{\alpha }:=\inf\{t>0:\frac{1+\sum_{j\in [p\cdot q]}I\{W_{j}\leq -t\}}{\max\{\sum_{j\in [p\cdot q]}I\{W_{j}\geq t\},1\}}\leq \alpha \}\;, \end{array}$$

where $T_{\alpha }>0$ is the knockoff threshold and α is the nominal FDR level. It can be shown that ${\hat{S}}_{\text{kn}}$ satisfies the FDR at level α, i.e. FDR ≤α (see Barber and Candès 2015, Candès *et al.* 2018 for further details). Note that we use the hat in ${\hat{S}}_{\text{kn}}$ to emphasize that the set of selected variables is the result of a random procedure. In literature, the knockoff threshold depicted in (9) is denoted as knockoffs+, a refined version of the standard knockoff method, introduced to provide stronger control over the FDR, where the number of negatives is incremented by 1. However, if being overly conservative is a concern, we can use the modified knockoff filter threshold $T_{\alpha }:=\inf\{t>0:\frac{\sum_{j\in [p\cdot q]}I\{W_{j}\leq -t\}}{\max\{\sum_{j\in [p\cdot q]}I\{W_{j}\geq t\},1\}}\leq \alpha \}$ which controls a modified version of the FDR. This adjustment helps increase the number of discoveries, particularly in the early stages of a study.

### 2.4 Derandomized multi-response knockoff procedure

Due to the inherent randomness of the standard MX knockoff filter, i.e. ${\hat{S}}_{\text{kn}}$ depends on a one-time construction of the stochastic knockoff copy $\tilde{Z}$, multiple runs of the MX knockoffs on the same dataset produce varying sets of selected variables, since for each run a new matrix of knockoffs is generated, which is not ideal in practice. To improve stability while preserving FDR control, we adopt a derandomized knockoff procedure, inspired by the aggregation approach of Ren and Barber (2024).

To ensure provable FDR control under arbitrary dependence structures, we recast the knockoff filter within the framework of *e*-values (Shafer *et al.* 2011, Wang and Ramdas 2022).

The aggregating knockoff procedure for FDR control takes advantage of one key property of the *e*-values, namely, the average of multiple *e*-values is still an *e*-value (Vovk and Wang 2021). Thus, given the initial dataset (Z,Y), we generate *M independent* knockoff copies ${\tilde{Z}}^{\left(1\right)},\ldots ,{\tilde{Z}}^{\left(M\right)}$ and then we compute $W_{j}^{\left(m\right)}$, the feature importance statistic related to the *m*th knockoff matrix. To perform knockoff selection on each copy at a target level β∈(0,1), for each m∈1,…,M, we define a knockoff threshold $T_{\beta }^{\left(m\right)}$ as follows:

(10) $$T_{\beta }^{\left(m\right)}=\inf\{t>0:\;\frac{1+\sum_{j\in [p\cdot q]}I\{W_{j}^{\left(m\right)}\leq -t\}}{\max\{1,\sum_{k\in [p\cdot q]}I\{W_{k}^{\left(m\right)}\geq t\}\}}\leq \beta \},$$

so that ${\hat{S}}_{\text{kn}}^{\left(m\right)}=\{j:W_{j}^{\left(m\right)}\geq T_{\beta }^{\left(m\right)}\}$ is the selected set for the knockoff filter when performed on the *m*th copy of the knockoff matrix ${\tilde{Z}}^{\left(m\right)}$. We define the *e*-values for j∈[p·q] as before

(11) $$e_{j}^{\left(m\right)}=\frac{pq\cdot I\{W_{j}^{\left(m\right)}\geq T_{\beta }^{\left(m\right)}\}}{1+\sum_{k\in [p\cdot q]}I\{W_{k}^{\left(m\right)}\leq -T_{\beta }^{\left(m\right)}\}}.$$

These *e*-values are then averaged across replicates:

(12) $$e_{j}^{\text{avg}}=\frac{1}{M}\sum_{m=1}^{M}e_{j}^{\left(m\right)}=\frac{1}{M}\sum_{m=1}^{M}{\text{weight}}_{j}^{\left(m\right)}I\{j\in {\hat{S}}_{\text{kn}}^{\left(m\right)}\},$$

where ${\text{weight}}_{j}^{\left(m\right)}$ corresponds to

$${\text{weight}}_{j}^{\left(m\right)}=pq\cdot \frac{1}{1+\sum_{k\in [p\cdot q]}I\{W_{k}^{\left(m\right)}\leq -T_{\beta }^{\left(m\right)}\}}\;.$$

Crucially, $e_{j}^{\text{avg}}$ is still a valid *e*-value due to the closedness of the *e*-value space under averaging. Finally, the e-BH procedure (Wang and Ramdas 2022) is applied to the averaged *e*-values at a target FDR level α, yielding the final selection set ${\hat{S}}_{\text{kn-derand}}$. This derandomized selection is more stable than the classical knockoff procedure, while maintaining finite-sample FDR control. Given a target level α, and for any choice of the parameter β∈(0,1) and any number of knockoff copies M≥1, the selected set ${\hat{S}}_{\text{kn-derand}}$, computed according to the proposed method, controls the FRD at level α. Ren and Barber (2024) provide the proof of this result and also discuss the optimal choice of β, recommending, for practical purposes, to fix β=α/2 when M>1 to achieve high power. Observe that when α=β and M=1, the derandomized procedure reduces to the original knockoff procedure at level α.

We briefly summarize the implemented procedure in the following Algorithm 2.

Algorithm 2 Derandomized Robust Multi-Response Knockoff filter (RobMRKF-Derand)

** Data**: n×q response matrix Y and n×p microbial features design matrix Z

** Parameters**  $\lambda_{1}$, $\lambda_{2}$, nominal FDR threshold α∈(0,1) and β, $M\in N_{+}$

** Procedure for**  m=1,…,M

** **1. sample the knockoff copy ${\tilde{Z}}^{\left(m\right)}$

** **2. model fitting according to Algorithm 1 on the augmented dataset $\left(Z,{\tilde{Z}}^{\left(m\right)},Y\right)$

** **3. compute the feature important statistics $W^{\left(m\right)}$ according to equation (8) (see Section 2.3 of the main paper)

** **4. compute the knockoff threshold $T^{\left(m\right)}$ according to (10)

** **5. compute the e-values $e_{j}^{\left(m\right)}$ according to (11)

** **6. **endfor**

** **7. compute the averaged e-values $e_{j}^{\text{avg}}=\frac{1}{M}\sum_{m=1}^{M}e_{j}^{\left(m\right)}$ for each j∈[p·q].

** **8. compute $\hat{\kappa }=\max\{\kappa :e_{\kappa }^{\text{avg}}\geq (pq)/(\alpha \kappa )\}$ or $\hat{\kappa }=0$ if this set is empty

** Output**: List of microbial features that are associated with at least one response variable, i.e. the selected set of discoveries ${\hat{S}}_{\text{kn-derand}}=\{j\in [p\cdot q]:e_{j}^{\text{avg}}\geq (pq)/(\alpha \hat{\kappa })\}$

Note that a non-robust version of Algorithm 2, named MRKF-Derand could be implemented by substituting Algorithm 1 in step 2 with the classical MRKF Algorithm (Srinivasan *et al.* 2023).

We have also deemed a variation of the RobMRKF-Derand, which consists of a two-step procedure: in the first step (*screening step*), a 10-fold cross-validation lasso procedure (Friedman *et al.* 2023) is adopted to face the very large dimensionality of the predictor space. For robust screening, we first remove multivariate outliers from the joined data of the response and the explanatory variables using the method of Filzmoser *et al.* (2008). In the second step (*selection step*), the RobMRKF-Derand is applied.

Further details on the theoretical background of *e*-values and the derandomized knockoff procedure are provided in Section 2.1, available as supplementary data at *Bioinformatics* online.

## 3 Simulation

We demonstrate the potential benefits of our *alr*RMLC method and the subsequent RobMRKF-Derand algorithm through extensive simulation studies. The knockoff method is implemented via the R-package knockoff (https://CRAN.R-project.org/package=knockoff).

### 3.1 Simulation settings

To mimic a real dataset that will be analyzed later in the paper, we consistently set n=100 and p=200 for all simulations.

In each replication of our simulation study, the rows of the n×p design matrix Z in (1) are randomly generated from a *p*-dimensional multivariate normal distribution with mean μ to be set as a vector of ones and covariance matrix $\Sigma =[\sigma_{jj^{\prime }}]$, where $\sigma_{jj^{\prime }}=0.5^{\left|j-j^{\prime }\right|}$, $1\leq j,j^{\prime }\leq p$. Note that this is equivalent to first generating microbiome relative abundances $\left(X_{1},\ldots ,X_{p+1}\right)$ through the logistic normal distribution $LN_{p}(\mu ,\Sigma )$ (Lin *et al.* 2014).

We randomly picked nonzero components in the regression coefficient matrix $\underset{p\times q}{B}$ by specifying a sparsity percentage ζ=10%, to allow for sparsity in the model. Next, each non-zero regression coefficient was randomly selected from {−3,−2,−1,1,2,3}. Finally, iid error terms $E_{j}$’s were simulated from $N_{q}(0,\Sigma )$ and the outcomes were calculated from Y=ZB+E.

To investigate whether the RobMRKF-Derand procedure (robust) and its classical counterpart (classical) are resistant to outliers, we considered three different scenarios:

- Scenario 1 (data without outliers): the design matrix Z and the responses Y are generated from multivariate normal distribution and the true model $\underset{n\times q}{Y}=\underset{n\times p}{Z}\underset{p\times q}{B}+\underset{n\times q}{E}$.
- Scenario 2 (data with outliers in the response only): the design matrix Z is generated as in Scenario 1. The response Y is generated according to the true model ($\underset{n\times q}{Y}=\underset{n\times p}{Z}\underset{p\times q}{B}+\underset{n\times q}{E}$), and then an ε=10% percentage of the observations are contaminated. That is, once rows of the error matrix E are simulated from a normal distribution, ε×n out of *n* randomly selected rows have random entries contaminated by numbers generated from $N_{q}(0,\gamma \times \Sigma )$.
- Scenario 3 (data with both outliers in the responses and in the covariates): $Z_{\mathrm{ori}}$ and the responses Y are generated as in Scenario 2. Then we replace the same ε percent observations of $Z_{\mathrm{ori}}$ as in Scenario 2 by outliers generated from a normal distribution $N_{p}(0,10\times \Sigma )$ and denote the new design matrix by Z.

For Scenario 1, we compared the efficiency of the proposed RobMRKF-Derand algorithm with the two-step procedure variation, as described earlier, with fixed values of p=200 and q=5 (classical versus classical with screening, and robust versus robust with screening). For the remaining scenarios, we always performed variable screening first.

For Scenario 2, we investigate:

- the effect of changing the number of dependent variables *q* (q=2,3,5,10), having fixed p=200;
- the effect of changing the magnitude of outliers, varying γ (γ=1,2,5,10,20)
- the effect of changing the sparsity ζ∈{2%,5%,10%,20%}

For Scenario 3, we investigate the effect of changing the percentage of outliers ε∈{0%,2%,5%,10%,20%}.

For every simulation setting, we created 100 replicated datasets, each with a sample size of n=100. The final selection set is computed via the derandomized knockoff filter run with the target nominal FDR level α = 0.2, drawing M=50 copies of the knockoffs. Note that we also modified the correlation parameter for the covariates, by considering $\sigma_{jj^{\prime }}=0.3^{\left|j-j^{\prime }\right|}$ and $\sigma_{jj^{\prime }}=0.7^{\left|j-j^{\prime }\right|}$, but the main findings are unchanged, and thus results are not explicitly presented here.

### 3.2 Performance evaluation and results

After generating the data, we used the RobMRKF-Derand algorithm (robust) and the non-robust MRKF-Derand (classical) to derive a sparse estimate of the regression coefficient matrix $\hat{B}$ and evaluated its accuracy by comparing it to the true coefficient B through the calculation of the proportion of false discoveries:

(13) $$\text{FDR}=\frac{\#\{j=1,\ldots ,p,h=1,\ldots ,q:{\hat{\beta }}_{jh}\neq 0\cap \beta_{jh}=0\}}{\#\{j=1,\ldots ,p,h=1,\ldots ,q:{\hat{\beta }}_{jh}\neq 0\}}$$

and the proportion of true positives:

(14) $$\text{TPR}=\frac{\#\{j=1,\ldots ,p,h=1,\ldots ,q:{\hat{\beta }}_{jh}\neq 0\cap \beta_{jh}\neq 0\}}{\#\{j=1,\ldots ,p,h=1,\ldots ,q:\beta_{jh}\neq 0\}}$$

We calculated the average value over 100 replications and termed the corresponding values as empirical FDR and empirical TPR, respectively, hereafter.

**Figure 1.**
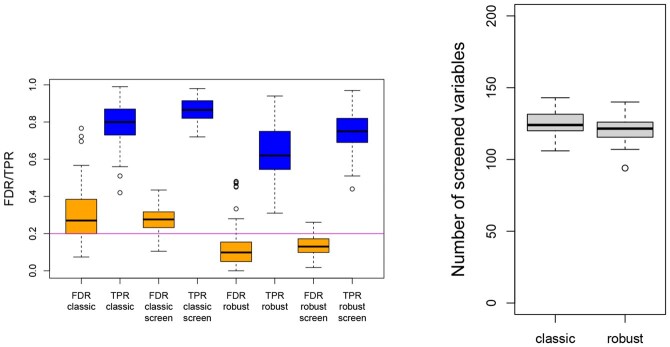
Simulation results for Scenario 1: comparison of classical and robust procedure, without and with variable screening (left), and the number of screened variables for the classical and robust method (right).

The results for Scenario 1 are presented in Fig. 1. The left plot shows all simulation results for FDR and TPR in terms of boxplots. We can observe that, in general, the two-step procedure, which includes an initial variable screening phase, leads to better performance in both approaches. The number of screened variables is relatively stable, see the right plot. Moreover, when comparing the classical and robust approaches, the latter undoubtedly demonstrates superior performance, as it ensures the FDR remains at the predetermined nominal level while also achieving higher power.

**Figure 2.**
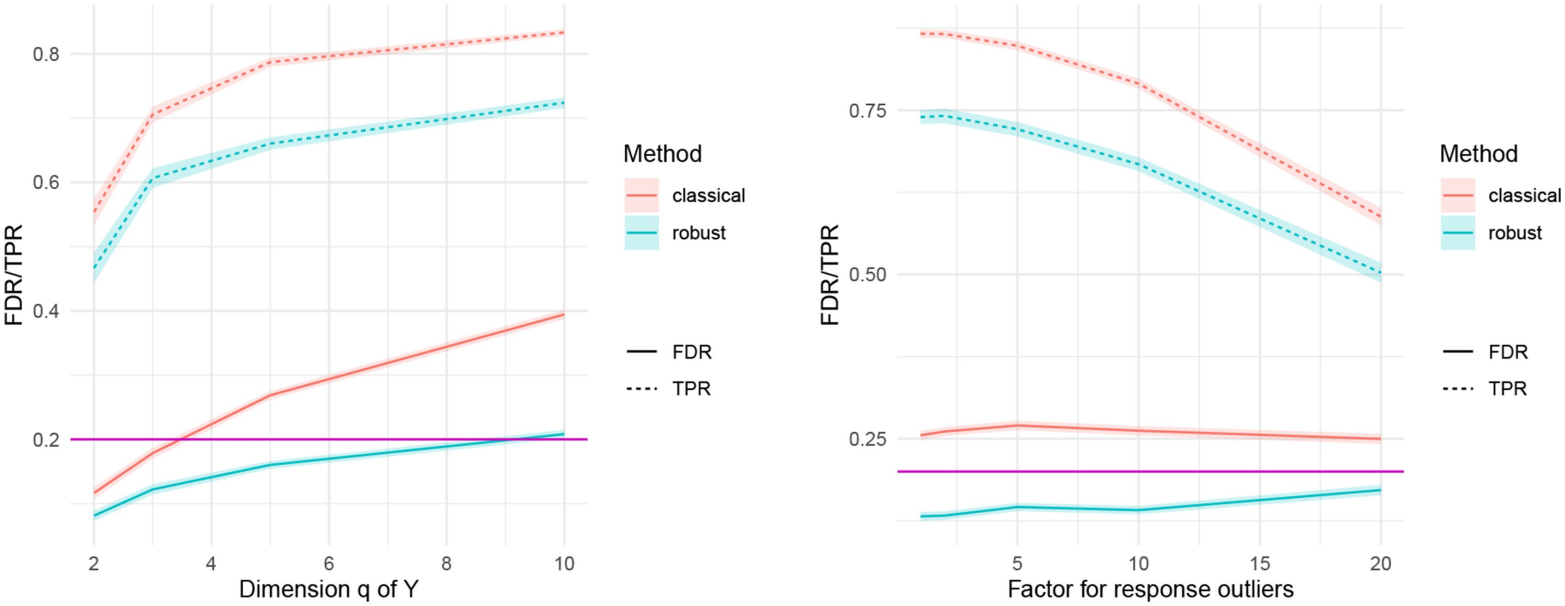
Simulation results for Scenario 2: comparison of classical and robust procedure, with varying dimension of the response (left), and the effect of varying the magnitude of the outliers by using γ=1, 2, 5, 10, 20 (right).

Figure 2 shows simulation results when outliers in the responses are present (Scenario 2). The plots show average FDR/TPR, plus/minus one standard error. As the response dimension *q* increases (left plot), the robust approach remains well centered around the nominal FDR value, while the classical method drifts dramatically. The price to pay is a lower power of the robust method compared to the classical method, although it still settles at acceptable levels. The effect of increasing the magnitude of the outliers γ (right plot) has no influence on maintaining the FDR, which remains nearly constant and below the nominal level for the robust method, but above the 20% level for the classical method. The TPR generally decreases with stronger outliers in the response space, and again, the robust method, maintaining the desired FDR level, shows a slight loss of power compared to the classical approach.

**Figure 3.**
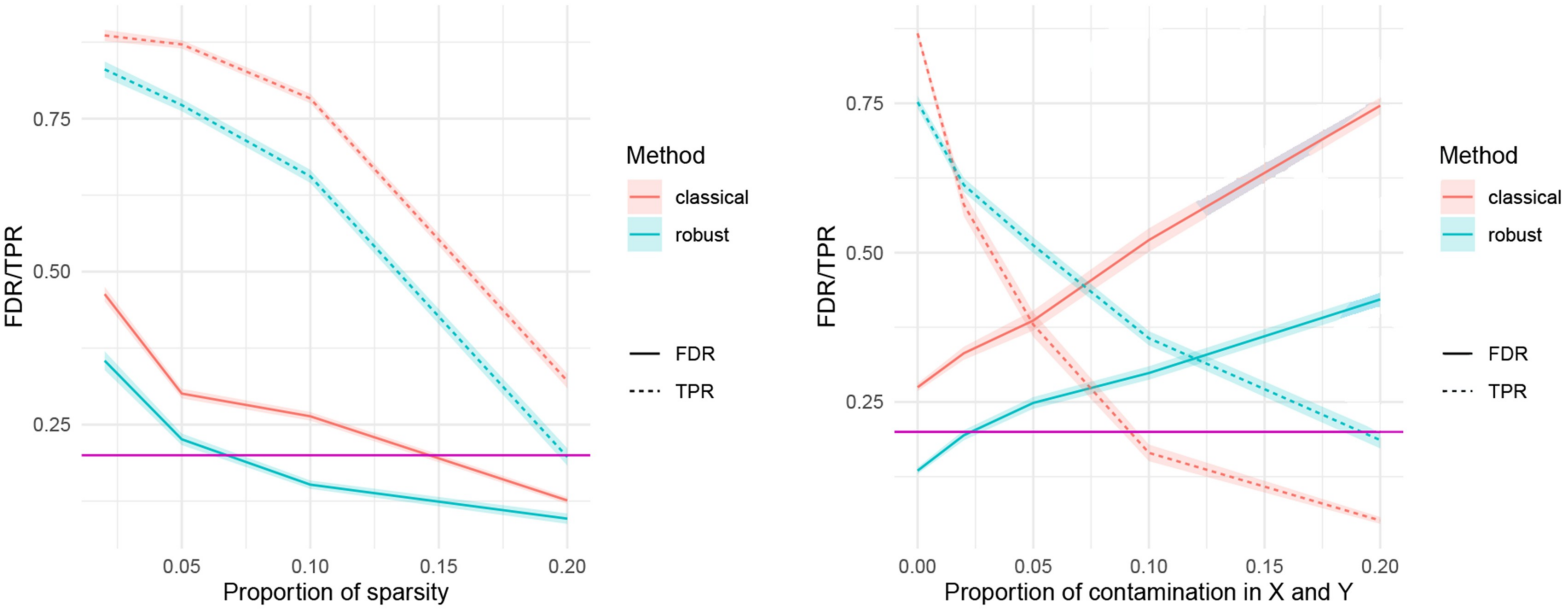
Simulation results comparing the classical and the robust procedure, with Scenario 2 by increasing the sparsity level (left), and Scenario 3 by increasing the amount of contamination (right).

Increasing the level of signal sparsity has a positive effect on the empirical FDR; however, for both approaches, the empirical power decreases considerably [see Fig. 3 (left)].

When both the covariates and the responses are contaminated with outliers (Scenario 3), the simulation results in Fig. 3 (right) show that increasing the proportion of outliers has a negative effect on the performance of both approaches in maintaining acceptable FDR levels. However, the robust method performs better: in the extreme case of 20% contamination, the empirical FDR of the robust method remains below 0.4, while the classical method reaches 0.75. Regarding power, in this scenario, it is pretty evident that the presence of large amounts of outliers has a dramatic effect on the performance of the classical method, which is based on a quadratic loss function. In contrast, the robust method achieves higher power, although it is still affected as the fraction of outliers increases.

## 4 Real data application

We illustrate the utility of our proposed method using intestinal microbiome data of the European Women’s Study by Karlsson *et al.* (2013). Processed relative abundances at the genus level were obtained from the curatedMetagenomicData database (Pasolli *et al.* 2017). The high-dimensional and sparse metagenomic data were first aggregated at the genus level, resulting in a final dataset of 176 bacterial abundances for 145 women with different disease statuses: type-2 diabetes (T2D; n=53), impaired glucose tolerance (IGT; n=49), and normal glucose tolerance (n=43). Instead of working with this categorical variable (disease status), we will explore the association of microbiome compositions and a multivariate response composed of four indicators of altered lipid and glucose metabolism (BMI, triglycerides, HDL, C-peptide), and one inflammation marker (hs-CRP). Specifically, BMI, triglycerides, C-peptide, or hs-CRP are associated with obesity, diabetes, or cardiovascular diseases, while higher levels of high-density lipoprotein (HDL) are common among healthy individuals. Thus, these variables can be considered as proxies or surrogates for disease status when diagnosis is not available. Moreover, working with multivariate continuous variables instead of a univariate categorical response could be more informative for identifying relevant associations between microbiome and disease.

**Figure 4.**
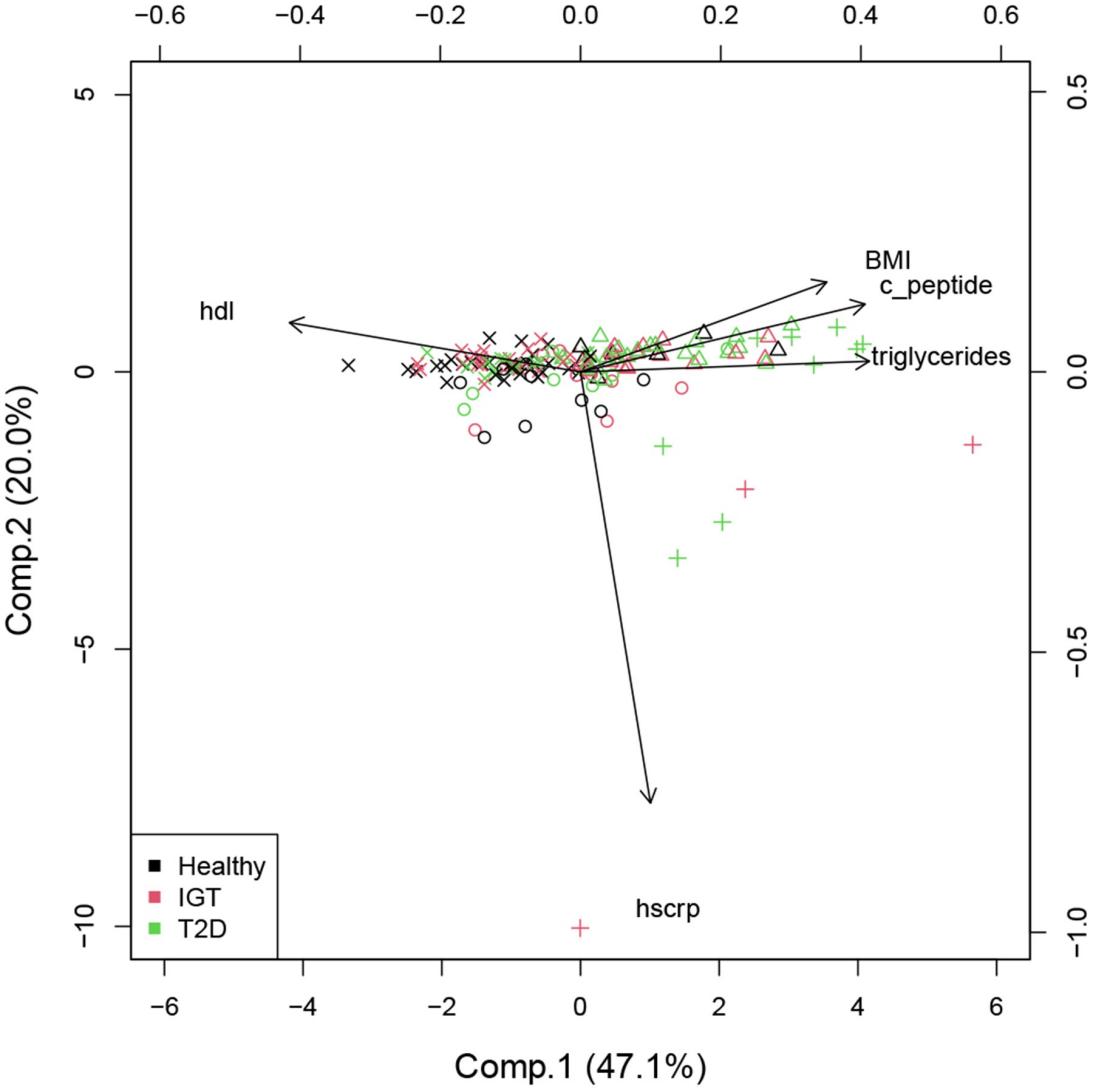
PCA biplot depicting the relationship between the dependent variables and the status of disease (see legend). The different symbols of the points represent the four clusters identified by model-based clustering.

Figure 4 illustrates the relationships between disease status and the five multivariate response variables (q=5) by presenting a biplot of a principal component analysis (PCA) of this response matrix. Here, the color information is according to the disease status, and the symbols are obtained by a cluster analysis of the response matrix, using model-based clustering, resulting in four clusters (Fraley and Raftery 2002). The first principal component, mainly defined by BMI, triglycerides, C-peptide versus HDL, shows discriminatory power between healthy and non-healthy subjects (IGT and T2D), explaining 47% of the total variance. The second component is defined by the inflammation marker hs-CRP. There are some potential outliers in the response matrix, mainly originating from the cluster encoded with the symbol “+”. This highlights the necessity of a robust data analysis.

The purpose of this application is to predict the five response variables using the microbiome composition as predictors. Due to very small abundances, we selected those genera that are present in at least 10% of the observations, resulting in p+1=100 bacterial genera. Further abundances reported as zero were replaced by random uniform numbers drawn from the interval $\left(0,x_{\min}\right)$, where $x_{\min}$ is the smallest value different from zero in the predictor matrix.

We consider the maximum association estimator (Alfons *et al.* 2017) to select the reference frame as described in Section 2 among the 100 possible candidates. Taking *Lactobacillus* as the alr reference, the predictor matrix now consists of p=99 alr variables, representing the bacterial genera. It is not surprising that a human gut commensal genus like, *Lactobacillus*, was chosen as alr reference. This lactic acid bacteria is widely present in the gut and maintains a mutualistic relationship with the human body, providing the host with dietary digestion and protection against pathogens in exchange for shelter and nutrients. *Lactobacillus* species are usually positively associated with good health and depleted in diseases like colon cancer, multiple sclerosis, HIV, and intestinal bowel disease. However, studies report contradictory results regarding their abundance in diabetes and obesity, which might be explained by the wide variety of metabolisms carried out by *Lactobacillus* species and strains (undetectable when working at the genus level) and the selection of one or another in every specific situation (Heeney *et al.* 2018). This supports the importance of selecting an alr reference as being dataset-specific.

In line with exploratory objectives and common practice in similar studies (Barber and Candès 2015, Dai and Barber 2016), we adopted a target nominal FDR level α = 0.2 for variable selection. This choice facilitates the identification of a broader set of candidate associations for further investigation. The RobMRKF-Derand algorithm selected a total of 7 bacterial genera predictive of the multivariate response. Note that the derandomization procedure applied to the MRKF algorithm in this example is entirely conservative, meaning that the final set is empty. Therefore, it is not possible to compare the two methods in this empirical example.

**Figure 5.**
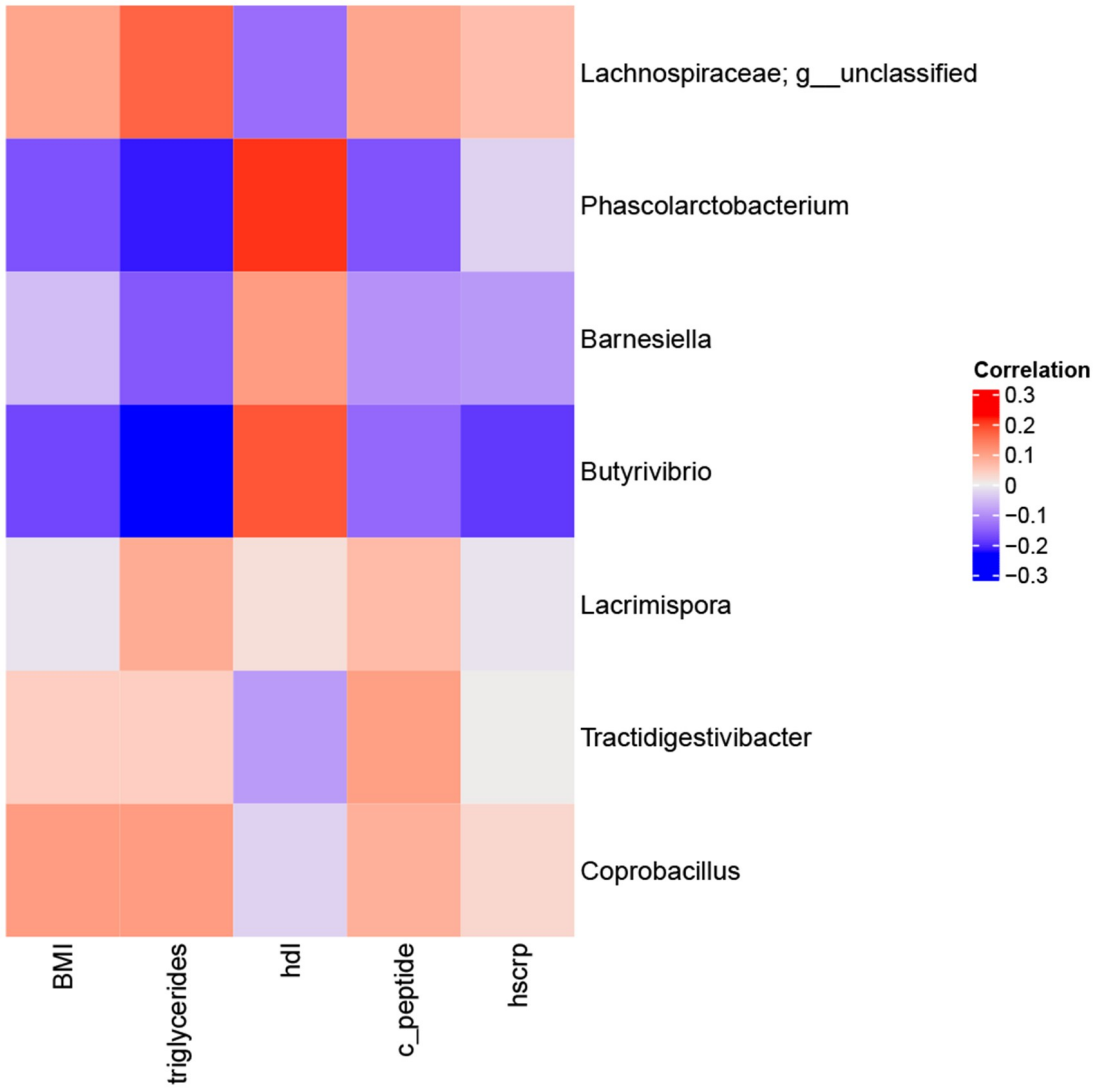
Spearman correlations heatmap.

To interpret the model, we computed Spearman correlations between every selected alr variable and the different response variables; see heatmap in Fig. 5.

Regarding the numerator part of the selected ALR, results show two main groups of bacteria that present opposite correlations regarding the health-associated biomarker response, HDL, and the rest of disease-associated biomarkers, i.e. BMI, triglycerides, and C-peptide.

On the one hand, *Phascolarctobacterium*, *Butyrivibrio*, and *Barnesiella*, which are known to be health-associated bacteria, correlate positively with HDL and negatively with the remaining response variables (BMI, triglycerides, and C-peptide). Both *Phascolarctobacterium* and *Butyrivibrio* are short-chain fatty acid producers, while *Barnesiella* presents anti-inflammatory properties (Vital *et al.* 2017). All of them have been previously reported to be abundant in healthy intestinal microbiomes compared to patients with T2D, hypercholesterolemia, and hypertension (Li *et al.* 2017, 2022, Granado-Serrano *et al.* 2019, Das *et al.* 2021, Hu *et al.* 2023).

On the other hand, a second group comprising unclassified genera from *Lachnospiraceae* family, *Tractidigestivibacter* and *Coprobacillus*, present opposite results: they correlate negatively with HDL and positively with BMI, triglycerides, and C-peptide. While the main producers of short-chain fatty acids in the human gut are genera from *Lachnospiraceae*, some of them are also associated with diseases affecting not only the gut but also peripheral organs. A review of different metagenomic studies reported an increase in *Lachnospiraceae* in subjects with metabolic disorders like obesity, diabetes, and non-alcoholic fatty liver disease (Vacca *et al.* 2020). In line with our results, Hu *et al.* (2023) also reported positive correlations between genera and species within *Lachnospiraceae* and several glucose and insulin homeostasis parameters, including fasting and postprandial C-peptide levels and insulin resistance estimators. *Coprobacillus* is also a common genus in human gut microbiome composition, and it has been associated with hypertension in mouse models (Li *et al.* 2017).

Additional diagnostic plots presented in the Supplementary Material (Fig. 1, available as supplementary data at *Bioinformatics* online) illustrate how the robust method effectively identifies outliers and leverage points that may influence estimation, thereby enhancing the reliability of the analysis.

## 5 Conclusions

This article introduces a robust knockoff filter for multivariate regression with compositional covariates, built on the e-BH procedure. The proposed method enhances the interpretability of variable selection while ensuring type I error control. Compared to the MRKF approach of Srinivasan *et al.* (2023), our method presents two key advancements. First, it incorporates a robust strategy to handle outliers in both predictors and responses, improving the stability of the selection process. Second, it introduces a derandomization step that reduces the variability in the final selection, ensuring greater reproducibility. This step is grounded in the strong connection between the knockoff framework and *e*-values, allowing us to reinterpret the knockoff filter as an e-BH procedure.

To properly account for the compositional nature of microbiome sequencing count data, we applied the additive logratio transformation. The alr requires selecting a reference taxon assumed not to be associated with the response. Although this assumption can be seen as a limitation, especially when the goal is to detect such associations, we argue that, in high-dimensional settings (with p≫n) and under sparsity assumptions (as in our Lasso-based regression framework), it is reasonable to expect that only a small subset of taxa are truly associated with the outcomes, making the existence of a “neutral” reference plausible. Moreover, the alr transformation offers clear interpretability of the results, unlike other log-ratio approaches such as ilr or clr, which—although theoretically well-founded—often produce results less directly linked to the original taxa.

We want to emphasize that in this paper, robustness refers to outlying observations in either the responses or in the covariates. This is the more traditional concept used in robust statistics (Maronna *et al.* 2019), while a more recent concept deals with outliers in single data cells (entries), which would be particularly attractive in the case of high-dimensional covariates (Raymaekers and Rousseeuw 2024). However, since cellwise robustness is not even available for the multivariate regression case, the combination with sparsity and compositional aspects is left as a topic of our future research.

The practical relevance of our method is illustrated using real microbiome data from individuals with varying glucose tolerance status. Even when disease classification is unavailable, individual health parameters may still capture underlying microbiome alterations. Our multivariate response approach reveals that such indicators are associated with the microbial signatures selected by the algorithm. This demonstrates the potential of our method in contexts where microbiome changes are better explained by continuous clinical markers rather than binary diagnoses. Both numerical simulations and real data applications confirm that the RobMRKF-Derand algorithm outperforms MRKF in the presence of outliers. Given the increasing relevance of multivariate regression with compositional covariates in microbiome research, our approach offers a robust and reproducible solution, advancing the statistical toolkit available for high-dimensional microbial data analysis.

## Supplementary Material

btaf506_Supplementary_Data

## Data Availability

The data underlying this article are available in Karlsson *et al.* (2013).
