# Supplementary material for: Robust multivariate regression controlling false discoveries for microbiome data
Source: Bioinformatics. 2025 Sep 17;41(9):btaf506. doi: 10.1093/bioinformatics/btaf506 (PMC12479396; doi:10.1093/bioinformatics/btaf506)
Supplement: btaf506_Supplementary_Data [file btaf506_supplementary_data.zip › BIOINF-2025-1553_SM.pdf]

Bioinformatics, 2022, pp. 1–4

doi: DOI HERE

Advance Access Publication Date: Day Month Year

Paper

PAPER

Supplementary Material for *Robust multivariate regression controlling false discoveries for microbiome data*

Gianna Serafina Monti,[1,*] Meritxell Pujolassos Tanyà,[2] Malu Calle Rosingana[2, 3] and Peter Filzmoser[3]

[1]Department of Economics, Management and Statistics, University of Milano-Bicocca, Piazza dell'Ateneo Nuovo, 1, 20126, Milan, Italy, [2]Bioscience Department, Faculty of Sciences, Technology and Engineering, University of Vic, Central University of Catalunya, 08500, Vic, Spain, [3]Institut de Recerca i Innovació en Ciències de la Vida i de la Salut, Catalunya Central (IRIS-CC), , 08500, Vic, Spain and [4]Institute of Statistics & Mathematical Methods in Economics, Vienna University of Technology, Wiedner Hauptstraße 8-10, 1040, Vienna, Austria

[*]Corresponding author. gianna.monti@unimib.it

FOR PUBLISHER ONLY Received on Date Month Year; revised on Date Month Year; accepted on Date Month Year

Abstract

S.1. Computational Details for alrRMLC Estimation

Complete algorithmic derivation, optimization strategies, and tuning parameter selection details for the proposed procedure are reported below. A summarized version of the final estimation algorithm is included in the main paper (Algorithm 1).

S.1.1. Solving $\mathbf{B}$ for a given $\boldsymbol{\Omega}$

In the first step, for a fixed precision matrix $\boldsymbol{\Omega}$ at a chosen point $\boldsymbol{\Omega}_0$, the objective function of the *alr*RMLC is optimized to get an estimate of $\mathbf{B}$, which is equivalent to a robust multivariate lasso regression (RML) with known covariance,

$$\widehat{\mathbf{B}}(\boldsymbol{\Omega}_0) = \arg\min_{\mathbf{B}} \Big\{ 2\sum_{i=1}^{n}\sum_{h=1}^{q} \rho\big([(\mathbf{Y}-\mathbf{ZB})\boldsymbol{\Omega}_0^{1/2}]_{ih}\big) + \lambda_2 \sum_{j=1}^{p}\sum_{h=1}^{q} |\beta_{jh}| \Big\}, \tag{S.1.1}$$

To solve the minimization problem (S.1.1) we implement an extension of the fast APG algorithm [Chang et al., 2018, Chang and Welsh, 2023] to adapt to the multivariate-response case with a fixed precision matrix to solve. An initial estimate of $\boldsymbol{\Omega}$ was estimated as the inverse of the residual covariance matrix, where residuals were obtained via cross-validated Lasso regressions of each response variable on the predictors.

In particular, problem (S.1.1) could be split into the sum of two separate parts: $f(\mathbf{B}) + g(\mathbf{B})$. The first one, $f(\mathbf{B}) = 2\sum_{i=1}^{n}\sum_{h=1}^{q}\rho\big([(\mathbf{Y}-\mathbf{ZB})\boldsymbol{\Omega}_0^{1/2}]_{ih}$, is differentiable, while the second one, $g(\mathbf{B}) = \lambda_2\sum_{j=1}^{p}\sum_{h=1}^{q}|\beta_{jh}|$ is non-differentiable as a lasso penalty is imposed on $\mathbf{B}$. The APG method [Beck and Teboulle, 2009] iterates $\mathbf{B}$ according to

$$\mathbf{B}_{k+1} = \text{prox}_{sg}(\mathbf{B}_k - s_k\nabla f(\mathbf{B}_k)),$$

where $\mathbf{B}_k$ is the kth iteration of $\mathbf{B}$, prox_{sg} is the proximal operator for g with a step size s, s_k is a suitably chosen step size at the kth iteration, and $\nabla f(\mathbf{B}_k)$ is the gradient of $f(\mathbf{B})$ evaluated at $\mathbf{B}_k$.

The proximal operator of g for the non-differentiable optimization part can be easily computed as it is the soft-thresholding operator [Chang and Welsh, 2023].

S.1.2. Solving $\boldsymbol{\Omega}$ for a given $\mathbf{B}$

In the second step, for a fixed $\mathbf{B}$ at a chosen point $\mathbf{B}_0$, the objective function of the *alr*RMLC is optimized to get an estimate of $\boldsymbol{\Omega}$ through a robust extension of the graphical lasso (Rglasso) [Yuan and Lin, 2007, Banerjee et al., 2008],

$$\widehat{\boldsymbol{\Omega}}(\mathbf{B}_0) = \arg\min_{\boldsymbol{\Omega}} \Big\{ 2\sum_{i=1}^{n}\sum_{h=1}^{q} \rho\big([(\mathbf{Y}-\mathbf{ZB}_0)\boldsymbol{\Omega}^{1/2}]_{ih}\big) - n\log|\boldsymbol{\Omega}| + \lambda_1 \sum_{j'\neq j} |\omega_{j'j}| \Big\}. \tag{S.1.2}$$

Note that the standard graphical lasso algorithm [Friedman et al., 2008] cannot be directly applied here because the objective function (S.1.2) has a non-standard sub-gradient. Analogous to Chang and Welsh [2023], we implement a robust extension of the graphical iterative shrinkage thresholding algorithm (G-ISTA) [Rolfs et al., 2012] to solve the graphical

© The Author 2022. Published by Oxford University Press. All rights reserved. For permissions, please e-mail: journals.permissions@oup.com

lasso, which uses a proximal gradient method for performing ℓ_1-regularized covariance matrix estimation.

S.1.3. Joint estimation of $\mathbf{B}$ and $\boldsymbol{\Omega}$

A 2-fold APG algorithm iterates between steps one and two until convergence to jointly estimate $\boldsymbol{\Omega}$ and $\mathbf{B}$. Convergence is reached when the reduction of the value of the objective function (S.1.2) from one iteration to the successive falls below some threshold. However, to enhance computational speed, a fast approximation — outlined in Algorithm 1 of the main paper— is adopted, which performs the outer iteration only once.

The tuning parameters λ_1 and λ_2 are chosen using K-fold cross-validated trimmed mean square prediction error (TMSPE), trimming the $\gamma\%$ of biggest squared residuals,

$$\text{TMSPE}_{\text{CV}}^{\lambda_1,\lambda_2} = \frac{1}{K}\sum_{\kappa=1}^{K}\frac{1}{n_\kappa q(1-\gamma)}\sum_{i=1}^{n_\kappa q(1-\gamma)}(\hat{\mathbf{y}}_{\lambda_1,\lambda_2}^{-k}-\mathbf{y}^k)^2_{(i)}\,, \tag{S.1.3}$$

where K is the number of folds, n_κ is the number of observations in the κth fold, $\hat{\mathbf{y}}_{\lambda_1,\lambda_2}^{-\kappa}$ defines an $n_\kappa q \times 1$ vector of the fitted responses computed using the data with the κth fold removed, $\mathbf{y}^\kappa$ is the observed responses of the κth fold of the data in an $n_\kappa q \times 1$ vector form, and $(.)^2_{(i)}$ denotes the set of ordered squared residuals in non-descending order.

S.2. Details on Multi-response Knockoff filter

The core concept behind knockoffs is to create 'artificial' covariates, i.e., the knockoff copies $\widetilde{\mathbf{Z}}$, which play the role of negative controls, whose distribution closely resembles that of the true covariates. Considering only $\mathbf{Z}$ and $\widetilde{\mathbf{Z}}$, it is impossible to determine whether the jth column represents a true variable or its knockoff copy; however, by examining $\mathbf{Y}$, we can ascertain if Z_j is non-null.

The knockoff copies preserve the dependence structure of the original variables and satisfy two key properties. First, they are pairwise exchangeable, meaning that for any subset $S_0 \subset \{1,\ldots,[p\cdot q]\}$ of null features, the joint distribution satisfies:

$$(\mathbf{Z},\widetilde{\mathbf{Z}})_{\text{swap}(S_0)}\mathbf{Y} \overset{d}{=} (\mathbf{Z},\widetilde{\mathbf{Z}})\mathbf{Y}\,, \tag{S.2.1}$$

where $\overset{d}{=}$ denotes equality in distribution, and the matrix $(\mathbf{Z},\widetilde{\mathbf{Z}})_{\text{swap}(S_0)}$ is obtained by swapping the entries Z_j and $\widetilde{Z}_j$ for each $j \in S_0$. Second, the knockoff variables are conditionally independent of the response, i.e., $\mathbf{Y} \perp\!\!\!\perp \widetilde{\mathbf{Z}}|\mathbf{Z}$, ensuring that they carry no additional information about the outcome and should not be associated with the response under the null. For further details on knockoff construction, see, e.g. Bates et al. [2021], Candès et al. [2018].

In the MX knockoff procedure, the marginal distribution of the covariates, i.e., the log-ratio variables $\mathbf{Z} = (Z_1,\ldots,Z_p)^{\text{T}}$, is supposed to be known, to construct knockoff copies obeying result (S.2.1). We consider $\mathbf{Z}$ following a multivariate Gaussian [Aitchison and Shen, 1980, Aitchison, 1982] distribution. Note that the alr transformation is a bijection; thus, this is equivalent to assuming that the random microbiome composition follows a logistic normal distribution [Aitchison and Shen, 1980]. No knowledge of the conditional distribution of the outcome $\mathbf{Y}$ given the covariates $\mathbf{Z}$ is required. We assume that the observations $(Z_{i1},\ldots,Z_{ip},Y_{i1},\ldots,Y_{iq}) \in \mathbb{R}^p \times \mathbb{R}^q$ are independently and identically distributed (iid), namely, the subjects are supposed to be a random sample from some arbitrary $(p+q)$-dimensional joint distribution $F_{\mathbf{ZY}}$. Then the MX knockoff matrix $\widetilde{\mathbf{Z}}$ is designed so that, for each observation label i $(\widetilde{Z}_{i1},\ldots\widetilde{Z}_{ip})$ serves as a knockoff for $(Z_{i1},\ldots Z_{ip})$, as described above.

To generate knockoff copies, we rely on the general and abstract framework of the Sequential Conditional Independent Pairs (SCIP) algorithm introduced by Candès et al. [2018], which ensures the creation of exchangeable knockoffs and applies to any probability distribution $F_{\mathbf{Z}}$. In this specific case, given the multivariate normality assumption of the microbial features $\mathbf{Z}$, we propose an approximate MX-knockoff construction based on second-order MX Gaussian knockoffs, designed to maintain the required exchangeability properties up to the first two moments of the joint distribution of $\mathbf{Z}$ and $\widetilde{\mathbf{Z}}$, rather than preserving them for the entire distribution. This approach yields valid knockoffs when $\mathbf{Z}$ follows a Gaussian distribution and serves as a good approximation when $\mathbf{Z}$ can be reasonably modeled as approximately normal [Barber et al., 2020]. We suggest considering a robust version of the second-order MX Gaussian knockoff construction, in which we robustly estimate from the data the mean and the covariance of the rows of $\mathbf{Z}$. In our implementation, we use the OGK estimator [Maronna and Zamar, 2002], a robust estimator of mean and covariance, which also works for data with more variables than observations.

S.2.1. Derandomized procedure

We generate M independent knockoff copies $\widetilde{\mathbf{Z}}^{(1)},\widetilde{\mathbf{Z}}^{(2)},\ldots,\widetilde{\mathbf{Z}}^{(M)}$, and for each run we apply the knockoff filter to $(\mathbf{Z},\widetilde{\mathbf{Z}}^{(m)},\mathbf{Y})$ $(m=1,\ldots,M)$ to obtain a selection set $\hat{S}_{\text{kn}}^{(m)}$ with FDR control. Then, for each $j \in [p\cdot q]$ we compute the weighted selection frequency

$$\Pi_j = \frac{1}{M}\sum_{m=1}^{M}\text{weight}_j^{(m)}\mathbb{I}\{j \in \hat{S}_{\text{kn}}^{(m)}\} \tag{S.2.2}$$

where $\text{weight}_j^{(m)}$ is a data-dependent weight, which is lower if, in the mth run, many features were selected. The final selection set is defined as $\hat{S}_{\text{kn-derand}} = \{j : \Pi_j \geq \text{a data-dependent threshold}\}$. The FDR of $\hat{S}_{\text{kn-derand}}$ is controlled.

The key for the FDR control is to use the so-called e-values [Shafer et al., 2011, Vovk and Wang, 2021, Wang and Ramdas, 2022], an alternative to the p-value, for quantifying evidence against the null hypothesis.

An e-value e for a null hypothesis $\mathcal{H}_0$ is a realization of a non-negative e-variable E which satisfies $\mathbb{E}_{\mathcal{H}_0}(E) \leq 1$. In contrast, we recall that a p-value for $\mathcal{H}_0$ is the realization of a p-variable P, such that $\mathbb{P}_{\mathcal{H}_0}(P \leq t) = t$, for all $t \in (0,1)$. Given a test of significance of level α, we reject $\mathcal{H}_0$ when $p \leq \alpha$, or equivalently, when $e \geq 1/\alpha$ as a consequence of Markov's inequality: $\mathbb{P}_{\mathcal{H}_0}(E \geq \frac{1}{\alpha}) = \alpha \cdot \mathbb{E}_{\mathcal{H}_0}(E) \leq \alpha$. In words: the larger the value of e, the stronger the evidence against the null hypothesis. E-values possess nice properties that p-values do not have. For example, if e_1 and e_2 are both e-values, $(e_1+e_2)/2$ is also an e-value (this is true regardless of the dependence structure of the e-values).

Suppose we want to test simultaneously multiple $p\cdot q$ hypotheses with $p\cdot q$ e-values $e_1,\ldots,e_{pq}$, the e-BH multiple testing procedure [Wang and Ramdas, 2022], a generalization of the Benjamini–Hochberg algorithm [Benjamini and Hochberg, 1995] with target FDR level α, consists in ranking the e-values from the largest to the smallest, $e_{(1)} \geq e_{(2)} \geq \ldots \geq e_{(pq)}$, and then define the rejection set, i.e., the selected set of discoveries,

as

$$\hat{S}_{\text{ebh}} = \left\{ j : e_j \geq \frac{pq}{\alpha \hat{k}} \right\}, \text{where } \hat{k} = \max \left\{ k \in [p \cdot q] : e_{(k)} \geq \frac{pq}{\alpha k} \right\}. \tag{S.2.3}$$

Note that in case $\hat{S}_{\text{ebh}} = \emptyset$, then, by convention, $\hat{k} = 0$. The e-BH procedure controls the FDR at level α even under arbitrary dependence structures among the e-values [Wang and Ramdas, 2022].

Notably, MX knockoffs can be written as an e-BH procedure with an appropriate class of e-values defined on the basis of knockoff feature importance statistics W_j and the knockoff threshold T_α as

$$e_j = \frac{pq \cdot \mathbb{I}\{W_j \geq T_\alpha\}}{1 + \sum_{k \in [p \cdot q]} \mathbb{I}\{W_k \leq -T_\alpha\}}, \tag{S.2.4}$$

Worth mentioning is that the e-values, defined in (S.2.4), satisfy the requirements for the e-BH procedure as they are non-negative and, by the property of knockoffs, we can see that

$$\sum_{j \in \mathcal{H}_0} \mathbb{E}[e_j] = pq\mathbb{E}\Big[\frac{\sum_{j \in \mathcal{H}_0} \mathbb{I}\{W_j \geq T_\alpha\}}{1 + \sum_{k \in [p \cdot q]} \mathbb{I}\{W_k \leq -T_\alpha\}}\Big] \leq$$

$$pq\mathbb{E}\Big[\frac{\sum_{j \in \mathcal{H}_0} \mathbb{I}\{W_j \geq T_\alpha\}}{1 + \sum_{k \in \mathcal{H}_0} \mathbb{I}\{W_k \leq -T_\alpha\}}\Big] \leq pq$$

Interestingly, it can be shown that $\hat{S}_{\text{kn}}$, the set of selected features for the knockoff procedure (see Section 2.3 of the main paper, equation (9)), and $\hat{S}_{\text{ebh}}$, the set of selected features for the e-BH procedure (S.2.3) applied to $e_1, \ldots, e_{pq}$ defined in (S.2.4) coincide [Ren and Barber, 2024]. It means that applying an e-BH procedure to the e-values defined in (S.2.4) recovers the knockoff procedure.

S.3. Real Data Example: Diagnostic Plots and Outlier Detection

Figure S.1 presents diagnostic plots. For that purpose, we compute the residual matrix from the final robust model, center column-wise by the median, and compute Mahalanobis distances with respect to the robustly estimated (inverse) matrix $\boldsymbol{\Omega}$. Similarly, we estimate a regression model of the response matrix on the (robustly) selected predictors using the least-squares estimator, compute the residual matrix, mean-center column-wise, and calculate Mahalanobis distances with respect to a classical estimate of the covariance matrix of the residuals. The left plot displays the Mahalanobis distances of the robust residuals versus those of the classical residuals, and the horizontal and vertical lines present the standard outlier cutoff value $\sqrt{\chi^2_{q,0.975}}$, equal to 3.582 [Rousseeuw and Driessen, 1999]. We use the same colors and symbols as in Figure 4 in the main paper. It can be seen that the strong outliers are identified by both the robust and the classical methods. However, for the classical method, we used the variable set identified by the robust method, as the classical procedure did not yield results on its own. These outliers exhibit increased abundance in hs-CRP; see Figure 4 in the main paper.

A further diagnostic plot is shown in Figure S.1 right. It shows robust Mahalanobis distances of the selected final set of alr-transformed genera, based on the MCD estimator [Rousseeuw and Driessen, 1999], against the Mahalanobis distances of the robust residuals. This plot combines information about regression outliers and leverage points, providing more insight than either distance metric separately. The vertical line represents the cutoff value $\sqrt{\chi^2_{|\hat{S}_{\text{kn-derand}}|,0.975}}$, equal to 4.00, since $|\hat{S}_{\text{kn-derand}}| = 7$. Colors and symbols are selected as before, and several of the + symbols are outliers in the response as well as in the residual space. Thus, they are bad leverage points [Rousseeuw and Driessen, 1999]. The robust approach clearly highlights the nature of these anomalous observations, allowing the researcher to conduct further diagnostic investigations. In contrast, with a classical method, we can assume that these anomalous values could be very influential to the analysis.

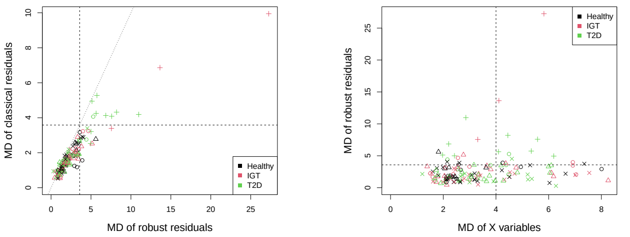

Fig. S.1. Plot of Mahalanobis distances of classical versus robust residuals (left), plot of Mahalanobis distances of robust residuals versus Mahalanobis distances of the alr-transformed selected taxa (right).

References

J. Aitchison. The statistical analysis of compositional data. *J R Stat Soc Series B Stat Methodol*, 44(2):139–177, 1982. doi: 10.1111/j.2517-6161.1982.tb01195.x.

J. Aitchison and S. M. Shen. Logistic-normal distributions: Some properties and uses. *Biometrika*, 67(2):261–272, 1980. doi: 10.2307/2335470.

O. Banerjee, L. El Ghaoui, and A. d'Aspremont. Model selection through sparse maximum likelihood estimation for multivariate gaussian or binary data. *J. Mach. Learn. Res.*, 9:485–516, 2008.

R. F. Barber, E. J. Candès, and R. J. Samworth. Robust inference with knockoffs. *Ann Stat*, 48(3):1409 – 1431, 2020. doi: 10.1214/19-AOS1852.

S. Bates, E. Candès, L. Janson, and W. Wang. Metropolized knockoff sampling. *J Am Stat Assoc*, 116(535):1413–1427, 2021. doi: 10.1080/01621459.2020.1729163.

A. Beck and M. Teboulle. Gradient-based algorithms with applications to signal-recovery problems. In D. P. Palomar and Y. C. Eldar, editors, *Convex Optimization in Signal Processing and Communications*, page 42–88. Cambridge University Press, 2009.

Y. Benjamini and Y. Hochberg. Controlling the false discovery rate: A practical and powerful approach to multiple testing. *J R Stat Soc Series B Stat Methodol*, 57(1):289–300, 1995. doi: 10.1111/j.2517-6161.1995.tb02031.x.

E. Candès, Y. Fan, L. Janson, and J. Lv. Panning for gold: 'model-X' knockoffs for high dimensional controlled variable selection. *J R Stat Soc Series B Stat Methodol*, 80(3):551–577, 2018. doi: 10.1111/rssb.12265.

L. Chang and A. H. Welsh. Robust multivariate lasso regression with covariance estimation. *J Comput Graph Stat*, 32(3): 961–973, 2023. doi: 10.1080/10618600.2022.2118752.

L. Chang, S. Roberts, and A. H. Welsh. Robust lasso regression using tukey's biweight criterion. *Technometrics*, 60(1):36–47, 2018. doi: 10.1080/00401706.2017.1305299.

J. Friedman, T. Hastie, and R. Tibshirani. Sparse inverse covariance estimation with the graphical lasso. *Biostatistics*, 9(3):432–441, 2008. doi: 10.1093/biostatistics/kxm045.

R. A. Maronna and R. H. Zamar. Robust estimates of location and dispersion for high-dimensional datasets. *Technometrics*, 44(4):307–317, 2002.

Z. Ren and R. F. Barber. Derandomised knockoffs: leveraging e-values for false discovery rate control. *J R Stat Soc Series B Stat Methodol*, 86(1):122–154, 2024. doi: 10.1093/jrsssb/qkad085.

B. Rolfs, B. Rajaratnam, D. Guillot, I. Wong, and A. Maleki. Iterative thresholding algorithm for sparse inverse covariance estimation. In F. Pereira, C. Burges, L. Bottou, and K. Weinberger, editors, *Advances in Neural Information Processing Systems*, volume 25. Curran Associates, Inc., 2012.

P. J. Rousseeuw and K. V. Driessen. A fast algorithm for the minimum covariance determinant estimator. *Technometrics*, 41(3):212–223, 1999. doi: 10.2307/1270566.

G. Shafer, A. Shen, N. Vereshchagin, and V. Vovk. Test Martingales, Bayes Factors and p-Values. *Statistical Science*, 26(1):84 – 101, 2011.

V. Vovk and R. Wang. E-values: Calibration, combination and applications. *Ann Stat*, 49(3):1736 – 1754, 2021. doi: 10.1214/20-AOS2020.

R. Wang and A. Ramdas. False Discovery Rate Control with E-values. *J R Stat Soc Series B Stat Methodol*, 84(3):822–852, 2022. doi: 10.1111/rssb.12489.

M. Yuan and Y. Lin. Model selection and estimation in the gaussian graphical model. *Biometrika*, 94(1):19–35, 2007.
